# Effect of intubation in the lateral position under general anesthesia induction on the position of double-lumen tube placement in patients undergoing unilateral video-assisted thoracic surgery: study protocol for a prospective, single-center, parallel group, randomized, controlled trial

**DOI:** 10.1186/s13063-023-07075-9

**Published:** 2023-01-29

**Authors:** Xi Zhang, Dong-Xu Wang, Qin Zhang, Qi-Bin Shen, Fei Tong, Yong-He Hu, Zhen-Duo Zhang, Fei-Fan Liu, Ya-Wen Tang, Juan-Li Chen, He Liu, Feng Zhou, Si-Ping Hu

**Affiliations:** 1 Department of Anesthesiology & Huzhou Key Laboratory of Basic Research and Clinical Translation for Neuromodulation, Huzhou Central Hospital || The Affiliated Huzhou Hospital, Zhejiang University School of Medicine || Affiliated Central Hospital Huzhou University, No. 1558 Sanhuan North Road, Wuxing District, Huzhou, Zhejiang Province 313000 China; 2 Department of Anesthesiology, The First Affiliated Hospital, Zhejiang University School of Medicine, Hangzhou, China; 3 Department of Cardiothoracic Surgery, Huzhou Central Hospital || The Affiliated Huzhou Hospital, Zhejiang University School of Medicine || Affiliated Central Hospital Huzhou University, No. 1558 Sanhuan North Road, Wuxing District, Huzhou, Zhejiang Province 313000 China; 4 Department of Operating Room Nursing, Huzhou Central Hospital || The Affiliated Huzhou Hospital, Zhejiang University School of Medicine || Affiliated Central Hospital Huzhou University, No. 1558 Sanhuan North Road, Wuxing District, Huzhou, Zhejiang Province 313000 China; 5 Medical Department, Huzhou Central Hospital || The Affiliated Huzhou Hospital, Zhejiang University School of Medicine || Affiliated Central Hospital Huzhou University, No. 1558 Sanhuan North Road, Wuxing District, Huzhou, Zhejiang Province 313000 China

**Keywords:** Double-lumen tube, Intratracheal intubation, Malposition, Lateral position, Video-assisted thoracic surgery, Thoracic anesthesia, Fiberoptic bronchoscopy

## Abstract

**Background:**

The double-lumen tube (DLT) is an essential equipment for thoracic anesthesia and the precise position of DLT placement is particularly important for anesthesia and surgery. However, the incidence of DLT malposition remains high and it leads to lung isolation failure and hypoxemia during one-lung ventilation. This trial aims to explore the clinical application and efficacy of intubation in the lateral position under general anesthesia induction to reduce the incidence of DLT malposition in patients undergoing unilateral video-assisted thoracic surgery (VATS).

**Methods:**

In this prospective, single-center, parallel group, randomized, controlled trial, we will recruit 108 patients, aged 18–80 years, scheduled for elective unilateral VATS with DLT intubation under general anesthesia, and they will be randomly assigned to two groups: a lateral DLT intubation group (group L) and a conventional supine DLT intubation group (group C). The left-sided DLT will be used to intubate in patients of both groups. The position of DLT will be confirmed and adjusted by using the fiberoptic bronchoscopy (FOB). The primary outcome is the incidence of DLT malposition observed via the FOB, and the secondary outcomes include the time of intubation, the frequency and duration of re-adjustments of DLT placement under FOB, whether to re-intubate, intraoperative vital signs, and postoperative recovery.

**Discussion:**

Accurate DLT positioning is crucially important for thoracic surgery, but the incidence of DLT malposition is still high in the present clinical practice of thoracic anesthesia. This trial aims to investigate whether lateral DLT intubation can reduce the incidence of DLT malposition, with more stable intraoperative vital signs and less postoperative complications.

**Trial registration:**

The study protocol was registered at Chinese Clinical Trial Registry (http://www.chictr.org.cn) with registration number: ChiCTR2200060794 on June 11, 2022.

**Supplementary Information:**

The online version contains supplementary material available at 10.1186/s13063-023-07075-9.

## Administrative information

**Table:** 

Title {1}	Effect of intubation in the lateral position under general anesthesia induction on the position of double-lumen tube placement in patients undergoing unilateral video-assisted thoracic surgery: study protocol for a prospective, single-center, parallel group, randomized, controlled trial
Trial registration {2a and 2b}	Chinese Clinical Trial Registry (http://www.chictr.org.cn), Registration number: ChiCTR2200060794. Registered on June 11, 2022
Protocol version {3}	Protocol Version 5.0 dated 25–04-2022
Funding {4}	This trial is supported in part by grants from the Medical Science and Technology Project of Zhejiang Province (grant number: 2020ZH043), Huzhou Municipal Science and Technology Bureau (2020GY40 to SPH), Zhejiang Provincial Natural Science Foundation (LY21H090001 to SPH, LY22H090019 to HL), and National Natural Science Foundation of China (NSFC81300957 and NSFC82171227 to HL).
Author details {5a}	Xi Zhang (XZ)^1,2^, Dong-Xu Wang (DXW)^1^, Qin Zhang (QZ)^1^, Qi-Bin Shen (QBS)^3^, Fei Tong (FT)^1^, Yong-He Hu (YHH)^1^, Zhen-Duo Zhang (ZDZ)^1^, Fei-Fan Liu (FFL)^1^, Ya-Wen Tang (YWT)^1^, Juan-Li Chen (JLC)^4^, He Liu (HL)^1^*, Feng Zhou (FZ)^5^*, Si-Ping Hu (SPH)^1^* ^1^ Department of Anesthesiology & Huzhou Key Laboratory of Basic Research and Clinical Translation for Neuromodulation, Huzhou Central Hospital || The Affiliated Huzhou Hospital, Zhejiang University School of Medicine || Affiliated Central Hospital Huzhou University, Huzhou, China; ^2^ Department of Anesthesiology, The First Affiliated Hospital, Zhejiang University School of Medicine, Hangzhou, China ^3^ Department of Cardiothoracic Surgery, Huzhou Central Hospital || The Affiliated Huzhou Hospital, Zhejiang University School of Medicine || Affiliated Central Hospital Huzhou University, Huzhou, China; Huzhou, China; ^4^ Department of Operating Room Nursing, Huzhou Central Hospital || The Affiliated Huzhou Hospital, Zhejiang University School of Medicine || Affiliated Central Hospital Huzhou University, Huzhou, China; Huzhou, China; ^5^ Medical Department, Huzhou Central Hospital || The Affiliated Huzhou Hospital, Zhejiang University School of Medicine || Affiliated Central Hospital Huzhou University, No. 1558 Sanhuan North Road, Wuxing District, Huzhou 313,000, Zhejiang Province, China *Correspondence to Si-Ping Hu, Feng Zhou or He Liu **Roles and responsibilities:** Principal investigator: SPH and LH Study design: SPH and LH Trial coordination: SPH, FZ, JLC and QBS Collection of data: XZ, DXW, FT, YHH, ZDZ, FFL and YWT Postoperative follow-up: XZ and DXW Data analysis and interpretation: QZ Writing of the manuscript: SPH, LH and XZ
Name and contact information for the trial sponsor {5b}	Si-Ping Hu, Huzhou Central Hospital, Huzhou, China E-mail: hsp0526@163.com
Role of sponsor {5c}	This is an investigator-initiated trial. Si-Ping Hu and He Liu are the sponsors of this study, and are fully involved in planning and designing the study. The study design, data collection, data analysis, data interpretation, manuscript writing or publication decisions are conducted independently from the funding agency

## Introduction

### Background and rationale {6a}

The thoracic surgery has drastically increased in recent years, especially in the light of the outbreak of coronavirus diseases 2019. Routine “passive” computed tomography of the chest screening of inpatients timely detects some pulmonary disease requiring thoracic surgeries. With the advantages of less damage, minimizing complications, and quick postoperative recovery, the video-assisted thoracic surgery (VATS) has been the standard approach of treatment in most thoracic surgery [1]. The double-lumen tube (DLT) intubation can achieve lung isolation and one-lung ventilation (OLV) which are the basis of modern thoracic anesthesia and surgery [2, 3]. The accurate position of DLT placement can effectively isolate the ventilation pathways of both lungs to ventilate separately at the bronchial level, resulting in full atrophy of the operated lung and good exposure of the operative field to facilitate surgical operations, also preventing pus, bronchial secretions, or blood from the operated lung entering the healthy lung [2]. However, many reasons lead to DLT malposition which deviates from the accurate position, generally including neck flexion or extension during shifting the patient from supine to lateral position, surgical manipulation, and coughing [4–8]. Related to differences in DLT types, intubation methods, or definitions of malposition, the incidence of DLT malposition mainly varied in 26–37% [9–11], and about 40% of DLT-related complications are due to DLT malposition, including poor lung isolation, hypoxemia during OLV, atelectasis, and severely impaired ventilation, which can cause hypoxia, gas trapping, atelectasis, tension pneumothorax, cross-contamination and infections, and interference with surgical procedures [9, 12, 13]; thus, the accurate position of DLT placement plays a crucially important role in thoracic anesthesia. Knowing how to both properly position a DLT and correct a malpositioned DLT is significant for airway management during thoracic surgery and in other cases where single-lung isolation is necessary. The fiberoptic bronchoscopy (FOB) is the most effective and reliable method to confirm and adjust the DLT position, and after assessing with FOB, studies have revealed that DLT becomes malpositioned in 25 to 54% of cases, requiring prompt repositioning [3, 14–17]. Even if initial DLT placement is performed successfully, DLT malpositioning commonly occurs, as blind intubation and lateral positioning of the patient can often displace the DLT.

Passive neck flexion or extension movement of patients from supine to lateral position after DLT intubation are likely to lead to DLT malposition, and a recent study reported that shifting patients to lateral position increased endobronchial cuff pressure due to changes in gravity and the curvature or length of the left main bronchus [18]. Thus, we hypothesize it will be possible to assist patients undergoing VATS in the surgically required lateral position before induction of anesthesia, and then to carry out DLT intubation under general anesthesia induction, which can directly avoid the possible adverse effects of lateral positioning. After reviewing related articles, less references of lateral DLT intubation can be found. Martinez et al. [19] mentioned in their article that an important and unique issue for non-intubated thoracic surgery (NIVATS) was the training of lateral position intubation for emergencies, as patients in NIVATS were likely to require to DLT intubation, and lateral intubation was not difficult according to their experiences. In addition, Ajimi et al. [20] reported a case of successful left-sided DLT intubation in the right lateral position for a patient with a giant mediastinal tumor with tracheal compression, and OLV was performed safely during thoracic surgery without ventilatory failure or hypoxemia. Therefore, we design this trial to explore the clinical application and efficacy of intubation in the lateral position under general anesthesia induction, and indicate it can reduce the incidence of DLT malposition in patients undergoing unilateral VATS.

### Objectives {7}

We will conduct a prospective, single-center, parallel group, randomized, controlled trial to explore the effect of intubation in the lateral position under general anesthesia induction on the position of DLT placement in patients undergoing unilateral VATS. We will study whether the DLT intubation in the lateral position under general anesthesia induction can reduce the incidence of DLT malposition with more stable intraoperative vital signs and fewer postoperative complications, compared with the conventional supine DLT intubation.

### Trial design {8}

This study will be conducted as a prospective, single-center, parallel group, randomized, controlled trial.

## Methods: participants, interventions, and outcomes

### Study setting {9}

The study will be conducted in patients undergoing elective unilateral VATS with DLT intubation in Huzhou Central Hospital, Zhejiang, China.

### Eligibility criteria {10}

The inclusion and exclusion criteria for participants in this study are as follows:

Inclusion criteria:

1. Scheduled to undergo the unilateral VATS with left-sided DLT intubation under general anesthesia
2. American Society of Anesthesiologists (ASA) score of I to III
3. Age 18–80 years with capacity
4. Agree to participate in this study and sign informed consent

Exclusion criteria:

1. Difficulty in intubation at preoperative assessment (body mass index > 30 kg/m^2^, limited neck movement, mouth opening < 3 cm or Mallampati III–IV grades)
2. Failure of multiple attempts to DLT intubate
3. Severe mental illness and communication difficulties
4. Refusal to sign informed consent
5. History of pulmonary surgery

### Who will take informed consent? {26a}

Appropriate participants will be jointly identified by the chief physician of anesthesia and cardiothoracic surgery at our hospital. We will give the participants sufficient time to consider and voluntarily choose to participate in the study, then will obtain the written informed consent on the day before surgery from each participant of study.

### Additional consent provisions for collection and use of participant data and biological specimens {26b}

Before obtaining the participants' informed consent, we will explain the implement methods of lateral and supine DLT intubation, and account for the preoperative, intraoperative, and postoperative data needing to be collected. The specific content of methods and data will also be listed in the informed consent form. This study will not involve the collection of biological specimens.

### Interventions

#### Explanation for the choice of comparators {6b}

With the special position requirement of thoracic surgery, the patients often need to be shifted to the left or right lateral position after anesthesia and intubation. In thoracic anesthesia, the patients are placed conventionally in the supine position for induction and intubation, then be moved to the lateral position after completing the DLT positioning. Therefore, we chose the supine DLT intubation as the control.

### Intervention description {11a}

#### Preparing

All participants are routinely fasted for 8 h before surgery and routinely monitored with a 5-lead electrocardiogram, non-invasive blood pressure or invasive arterial blood pressure, and oxygen saturation in the operation room.

#### Description for intervention

Both groups will be induction and maintenance with total intravenous anesthesia, followed by the left-sided DLT (Broncho-Cath®, Mallinckrodt Medical Ltd., Hampshire, Ireland) intubation to mechanical assisted ventilation. Lateral DLT intubation group (group L): investigators assist the participant to a comfortable and surgically required lateral position before induction and intubate DLT with a video laryngoscope after induction, then confirm and adjust the DLT position using the FOB to complete the positioning of DLT. Conventional supine DLT intubation group (group C): investigators intubate DLT with a video laryngoscope after induction in the conventional supine position, then confirm and adjust the DLT position using the FOB to complete the positioning of DLT, and use the FOB again to confirm the DLT position after lateral positioning.

#### Introduction for investigators

This study will be completed with the joint participation of the Department of Anesthesiology, the Department of Cardiothoracic Surgery and the Department of Operating Room Nursing. For anesthesia, the investigator has extensive experience in thoracic anesthesia and has specially trained in lateral intubation.

#### Criteria for discontinuing or modifying allocated interventions {11b}

The investigator will terminate the experiment for this participant if one of the following occurs during the experiment:

1. Participants in the operating room not accept the intervention after learning about their grouping
2. Attempting DLT intubation in lateral position ≥ 3 times or intubation time ≥ 3 min
3. Switch to supine DLT intubation after unsuccessful attempts to lateral intubation
4. Postoperative follow-up could not be completed due to various reasons

#### Strategies to improve adherence to interventions {11c}

The principal investigator (SPH) will conduct the pre-anesthesia evaluation day before surgery, strictly following the exclusion and inclusion criteria. During obtaining informed consent from participants, SPH will explain the content of the study and the need for cooperation in detail for participants. Furthermore, another investigator will administer a brief questionnaire to participants for 3–5 min at 24 h postoperative without excessively affecting the rest time of participants.

#### Relevant concomitant care permitted or prohibited during the trial {11d}

All study participants will receive standard postoperative care in the operating room, the postanesthesia care unit (PACU), the intensive care unit (ICU), or the ward.

#### Provisions for post-trial care {30}

At the end of the intervention and surgery, participants will be extubated the DLT in the operating room and sent to the PACU for observation (or to the ICU with the DLT if the patient is in poor condition) and then sent back to the ward. There are risks associated with thoracic surgery and DLT intubation. Regardless of the reason for any adverse events, our department and the hospital will offer care.

### Outcomes {12}

#### Primary outcome

The incidence of DLT malposition observed via the FOB in lateral and supine intubation groups. The definition of DLT malposition is that the DLT is moved to correct its position by more than 1.0 cm [9].

#### Secondary outcomes

1. The time of intubation. Defined as the time from the use of the video-laryngoscope to confirm the correct position of the DLT by using the FOB
2. The frequency and duration of re-adjustments under FOB
3. Whether to re-intubate
4. Intraoperative vital signs, including heart rate, blood pressure and oxygen saturation
5. Postoperative recovery, including early postoperative complications and the Quality of Recovery-15 (QoR-15) questionnaire at 24 h after surgery

In designing this trial, we aim to explore the effect of lateral intubation after induction on the position of DLT in patients undergoing elective unilateral VATS. In order to visually and quantitatively measure the effect, the rate of DLT movement of more than 1.0 cm observed via the FOB will be compared between lateral intubation and supine intubation. This definition of DLT malposition was also used in the study by Inoue et al. as the authors indicated that Japanese patients were small and a deviation of 1.0 cm could be significant for them [9]. In China which is also in Asia, the DLT movement more than 1.0 cm is also critical for Chinese patients, so we select the deviation of 1.0 cm as the cut-off value.

For information on how to measure the distance of DLT movement, we refer to Desiderio et al. [8] on the method of measuring tracheal distance. After the DLT positioning is completed, the distance from the main carina to the distal tip of the tracheal lumen is measured in centimeters using the FOB as the carina-to-tracheal distance. Specifically, it is the difference between the depth when the tip of the FOB is at the main carina minus the depth when the tip of the FOB is at the distal tip of the tracheal lumen. The carina-to-tracheal distance measured after accurate DLT positioning is taken as the base value, and then measured again after turning to the lateral position or intraoperatively, a difference of more than 1.0 cm from the base value is recorded as DLT malposition.

#### Participant timeline {13}

**Fig. 1:**
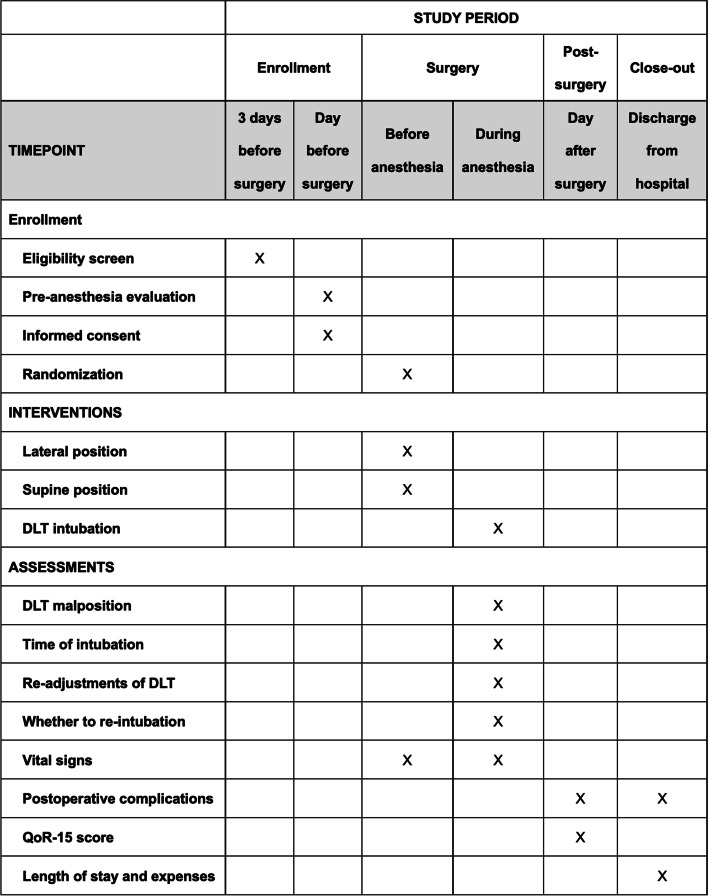
Time schedule of enrolment, interventions, assessments, and visits for participants. DLT, double-lumen tube; QoR-15, Quality of Recovery-15 questionnaire

The participant timeline is shown in Fig. 1.

#### Sample size {14}

This trial focuses on whether intubation in the lateral position under general anesthesia will reduce the DLT malposition rate observed via the FOB in patients proposed for unilateral VATS; therefore, the expected sample size is calculated based on the current DLT malposition rate. According to previous studies, the incidence of DLT malposition is about 30% [9]. Expecting a 50% reduction in malposition rate to be considered an effective intervention, we chose *α* = 0.05 and test efficacy *β* = 80%, which is calculated to yield 48 cases in each group by using the PASS 15.0 software. Considering a 10% drop-out rate, the final sample size is derived as at least 54 cases per group, with a total of 108 patients included.

#### Recruitment {15}

Study participants will be recruited from patients undergoing unilateral VATS at Huzhou Central Hospital. The research team including anesthesiologists and thoracic surgeons will involve in the recruitment process. There is an adequate source of patients to ensure the number of enrollment, and 4 months is enough to complete the enrollment of 108 eligible participants. Currently, this trial is in the recruitment phase and patients will be screened strictly according to the recruitment criteria.

### Assignment of interventions: allocation

#### Sequence generation {16a}

The computerized random number generator is used to generate random sequences on a 1:1 basis, and the random sequence will be put into a sealed, opaque and sequentially numbered envelope by the investigator. When the participant is admitted to the operating room the investigator will open the envelope to obtain a random sequence to determine the grouping. Participants with random sequences 1–54 are assigned to group L, while 55–108 are assigned to group C. Each participant will be given a unique study sequential number on the envelope cover in the order of their participation in this study. The random sequences of all participants and its corresponding study sequential numbers will be recorded in the randomization list.

#### Concealment mechanism {16b}

The random sequence, study sequential number and group allocation of participant will be typed onto separate pages and be collected to store confidentially. The randomization process will be performed by investigators who are not involved in postoperative follow-up and data analysis.

#### Implementation {16c}

The randomization stated above will decide the allocation of intubation position (lateral versus supine). Participants enter the operating room to be determined the grouping, then the investigator will inform the participant of the specific grouping and assist the participant in lateral or supine position. The principal investigator will prepare the envelope with random sequence for allocation.

### Assignment of interventions: blinding

#### Who will be blinded {17a}

This study is an open-label clinical trial. It is not possible to blind participants, anesthesiologists, and surgical team when the intervention is implemented in this trial. But for researchers in postoperative follow-up and data analysis, they will not know the allocation of participants.

#### Procedure for unblinding if needed {17b}

Unblinding will not occur because this study is an open-label clinical trial.

### Data collection and management

#### Plans for assessment and collection of outcomes {18a}

Data will be collected at three time points (preoperative, intraoperative and postoperative) and recorded on the case record form (CRF). The principal investigator (SPH) will conduct the pre-anesthesia evaluation and obtain informed consent from the participant day before surgery. Two anesthesiologists belonging to research team will implement the intervention, record intraoperative data, and complete the anesthesia in the operating room, with SPH as the emergency contact and involved in mentoring. Postoperative followers and data analysts will not be involved in the intervention or anesthesia process and will not know the grouping of participant.

The preoperative CRF will be completed by SPH on the day before surgery and consist mainly of basic patient information, airway assessment, and past medical history. The intraoperative CRF mainly records DLT malposition (time and measures of each malposition), vital signs, number of times and reasons for using FOB, etc. We define the DLT malposition as moving the DLT by more than 1.0 cm to correct its position [9]. The criteria for correct DLT position is defined via the FOB as follows: an unobstructed view into the left upper and lower lobe bronchus through the endobronchial lumen with the bronchial cuff directly below the carina and just visible in the main left bronchus through the tracheal lumen [14]. The postoperative CRF will be completed by the investigator not involved in the intervention and anesthesia process at 24 h postoperative and the day of discharging from hospital and focus on recording early postoperative complications and the QoR-15 score [21].

All collected data will be analyzed after the last participant has completed the trial by investigators not involved in the trial process. Compared to conventional supine intubation, we expect that lateral intubation will reduce the risks associated with DLT malposition, post-anesthetic position changes, and multiple uses of the FOB. Considering to ensure the safety and stability of the patient during surgery, the intraoperative vital signs and the incidence of hypoxemia will be investigated. Furthermore, we pay attention to the postoperative complications and the QoR-15 score which is more in line with the concept of enhanced recovery after surgery.

#### Plans to promote participant retention and complete follow-up {18b}

Considering that the intervention may not have long-term effects on participants, we will only perform the follow-up at 24 h postoperative. When obtaining informed consent, we will explain to the participant that there will be a brief questionnaire for 3–5 min and stress the importance of this.

#### Data management {19}

All participant data collected in this study will be processed in accordance with the Data Security Law of the People’s Republic of China. All participant data will be manually filled in the paper CRFs and then transcribed into Microsoft Excel by the researcher not involved in the implementation of the intervention. Primary monitoring will be performed by an independent research nurse, including checking all preoperative, intraoperative, and postoperative CRFs and informed consent forms for missing items, incorrect filling, and illegible writing. The coordinator will review all errors and verify that the errors are crossed out and finally annotated with a signature and date. In accordance with local and national regulations, raw data such as paper CRFs will be stored in a locked cabinet in the anesthesiology office that can only be opened by authorized personnel. Data entered into Microsoft Excel will be securely stored on a password-protected desktop computer in the locked office. Access is restricted to investigators assigned to data entry, processing, and analysis.

#### Confidentiality {27}

The randomization list and paper CRFs will be kept in a locked cabinet in the anesthesiology office under the supervision of the principal investigator. Considering that the paper CRFs contain participant names and study sequential numbers, access to the paper CRFs will be authorized only upon special request and after strict review, for the protection of participant privacy. De-identified data will be entered into Microsoft Excel, access codes will be set and stored on a locked computer, and only authorized investigators will have access. Any subsequent publications will not include any patient identifying information and study sequential numbers.

#### Plans for collection, laboratory evaluation, and storage of biological specimens for genetic or molecular analysis in this trial/future use {33}

Not applicable, there will be no biological specimens collected.

### Statistical methods

#### Statistical methods for primary and secondary outcomes {20a}

The SPSS statistical software version 26.0 (IBM) will be used for statistical analysis. Primary and secondary outcomes will be analyzed as follows: use Kolmogorov–Smirnov test to determine whether continuous data follows normal distribution. Continuous data following a normal distribution are expressed as mean ± standard deviation (‾x ± s), while nonnormal distributions are expressed as median (quartile spacing). The independent samples *t* test will be used for comparison between groups of normally distributed continuous data. The Mann–Whitney *U* test will be used for comparison of non-normally distributed continuous data. Count data are expressed as number of cases (rate) and tested by chi-square test or Fisher’s exact test. If subsequent multi-factor analysis will be required, multiple linear regression or logistic regression models are selected according to continuous-type or sub-type dependent variables. The data analysis of this study adopts the principle of intentional analysis. Multiple interpolation will be used for the analysis of missing data, and sensitivity analysis will be performed. Probability values < 0.05 will be considered significant.

#### Interim analyses {21b}

Not applicable, no interim analyses are planned.

#### Methods for additional analyses (e.g., subgroup analyses) {20b}

Not applicable, no additional analyses are planned.

#### Methods in analysis to handle protocol non-adherence and any statistical methods to handle missing data {20c}

Given that we will explain the intervention in detail and emphasize cooperation matters to the patient during pre-anesthesia evaluation and obtaining informed consent, we guess that few patients will protocol non-adherence. If needed, multiple interpolation will be used for the analysis of missing data, and sensitivity analysis will be performed.

#### Plans to give access to the full protocol, participant level-data, and statistical code {31c}

This is a principal investigator-initiated trial. Access to the full protocol and participant level data will be considered upon submission of a reasonable request and consent of the principal investigator.

### Oversight and monitoring

#### Composition of the coordinating center and trial steering committee {5d}

This is a single-center trial. No steering committee will be formed. Weekly group meetings will be initiated by the principal investigator to discuss the progress of the study.

#### Composition of the data monitoring committee, its role and reporting structure {21a}

We expect the rapidly inclusion of 108 participants and plan to complete the trial within 4 months; therefore, no data monitoring committee will be formed.

#### Adverse event reporting and harms {22}

Adverse events that occur in this trial will be recorded on the CRF and reported to the principal investigator.

Each adverse event will be assessed for the character (expected vs unexpected), severity (serious vs non-serious), and relevance to the intervention (relevant vs irrelevant). Serious and unexpected adverse events will be reported to the Ethics Committee. The principal investigator will conduct regular cumulative reviews of all adverse events and convene investigator meeting as necessary.

#### Frequency and plans for auditing trial conduct {23}

A research nurse who is not involved in the current trial will act as an independent reviewer for the duration of the trial. The audit process takes place every 2 weeks by the independent research nurse, and all errors will be recorded and reported to the principal investigator. The audit process will include a review of CRFs, registries, missing data, duplicate data, and informed consent documentation.

#### Plans for communicating important protocol amendments to relevant parties (e.g., trial participants, ethical committees) {25}

The Ethics Committee of Huzhou Central Hospital has reviewed the protocol and agreed to conduct the trial as the protocol. No protocol amendments will be made unless permitted by the Ethics Committee. Any important protocol modifications will be reviewed by the principal investigator who will sign the amendment, which will be submitted to the ethics committee for approval later.

#### Dissemination plans {31a}

Following statistical analysis of the trial, every endeavor will be made to publish the results in peer-reviewed journals related to clinical anesthesia and thoracic surgery.

## Discussion

There are many reasons for DLT malposition in thoracic surgery, especially shifting the patient from supine to lateral position which may lead to more malposition [8]. Several innovative approaches by many researchers have shown promising results in reducing the incidence of DLT malposition during lateral positioning. For example, it has been shown that the DLT malposition rate was lower in patients who removed the headrest before lateral positioning compared to those who used the headrest all the time [22]. In addition, one study found that limiting head and neck movements with a neck brace also minimized DLT malposition during supine to lateral position [7]. Unfortunately, these effective measures did not fundamentally solve the DLT malposition caused by the patient’s position change. We expect that the lateral intubation after assisting the patient to a comfortable and surgically required lateral position before induction will thoroughly resolve the DLT malposition or other adverse effects caused by lateral positioning. Of course, there is more than one cause for the malposition. All occurrences of DLT malposition will be recorded throughout the trial, as well the time and the possible reason.

For anesthesiologists who have never tried or even heard of lateral DLT intubation, this procedure seems awkward and difficult. However, based on our preliminary pre-experiments, this process is not as challenging as anticipated when using flexible intubation techniques and proper DLT shaping. After intubation in the lateral position, the patient does not need to be moved again and the operation can be started directly. It reduces the need for medical staff to shift position of patient under anesthesia, which is an unsafe and labor-intensive process, so the lateral DLT intubation is well received by operating room nurses and surgeons. The results of the study, we anticipate, will provide evidence for the clinical application and efficacy of lateral intubation, pioneering a new approach to DLT intubation in thoracic surgery.

## Trial status

The trial is registered on the Chinese Clinical Trial Registry (http://www.chictr.org.cn) with registration number: ChiCTR2200060794. The current protocol is version 5.0 of 25/04/2022. Recruitment for the trial starts in August 2022, and we are currently recruiting patients. Approximate date of recruitment completion is December 2022.

## Supplementary Information

**Additional file 1. **Preoperative, intraoperative and postoperative CRFs.

**Additional file 2. **QoR-15 score.

**Additional file 3. **Informed consent form.

## Data Availability

All data during the study are available from the corresponding author upon reasonable request.

## Abbreviations

ASA score: American Society of Anesthesiologists Score
CRF: Case record form
DLT: Double-lumen tube
FOB: Fiberoptic bronchoscopy
Group L: Lateral DLT intubation group
Group C: Conventional supine DLT intubation group
ICU: Intensive care unit
NIVATS: Non-intubated thoracic surgery
OLV: One-lung ventilation
QoR-15: Quality of Recovery-15
PACU: Postanesthesia care unit
VATS: Video-assisted thoracic surgery
