# Supplementary material for: Effect of intubation in the lateral position under general anesthesia induction on the position of double-lumen tube placement in patients undergoing unilateral video-assisted thoracic surgery: study protocol for a prospective, single-center, parallel group, randomized, controlled trial
Source: Trials. 2023 Jan 29;24:67. doi: 10.1186/s13063-023-07075-9 (PMC9884328; doi:10.1186/s13063-023-07075-9)
Supplement: Supplementary file 1 — Additional file 1. Preoperative, intraoperative and postoperative CRFs. [file 13063_2023_7075_MOESM1_ESM.pdf]

Preoperative CRF Name:______ Inpatient number:______

<table>
<tr><td>Gender</td><td></td><td rowspan="3">ASA</td><td>I</td><td></td></tr>
<tr><td>Age</td><td></td><td>II</td><td></td></tr>
<tr><td>Inpatient number</td><td></td><td>III</td><td></td></tr>
<tr><td>Radiation number</td><td></td><td rowspan="2">Smoking habits</td><td>Smoking duration and volume</td><td></td></tr>
<tr><td>Contact information 1</td><td></td><td>Give up smoking</td><td></td></tr>
<tr><td>Contact information 2</td><td></td><td rowspan="6">Recent situation</td><td>NSAIDs</td><td></td></tr>
<tr><td>Home address</td><td></td><td>Steroid</td><td></td></tr>
<tr><td>Weight/height (cm/kg)</td><td></td><td>Opioids</td><td></td></tr>
<tr><td>Preoperative diagnosis</td><td></td><td>Pharyngitis</td><td></td></tr>
<tr><td>Clinical stages</td><td></td><td>Gastroesophageal reflux disease</td><td></td></tr>
<tr><td>FVC（% of prediction）</td><td></td><td>Upper respiratory infections</td><td></td></tr>
<tr><td>FEV_1（% of prediction）</td><td></td><td rowspan="5">Patient special</td><td>Hypertension</td><td></td></tr>
<tr><td rowspan="2">Mallampatti classification</td><td>I</td><td>Diabetes</td><td></td></tr>
<tr><td>II</td><td>Cardiovascular disease</td><td></td></tr>
<tr><td>Presence of drug allergy</td><td></td><td>Chronic bronchitis</td><td></td></tr>
<tr><td rowspan="2">Whether suspicious difficult airway</td><td>Mouth opening limitation</td><td>Previous surgery</td><td></td></tr>
<tr><td>Cervical spondylopathy</td><td>Intended surgery</td><td colspan="2"></td></tr>
</table>

CRF: case record form; ASA score: American Society of Anesthesiologists Score; cm: centimeter; kg: kilogram; FVC: forced vital capacity; FEV_1: forced expiratory volume in 1 second; NSAIDs: non-steroidal anti-inflammatory drugs.

Intraoperative CRF   Name:______   Inpatient number:______

Anesthesia duration (min)		
Bleeding (ml)		
Urine volume (ml)		
Infusion type and total (ml)		
DLT	Model	
	Intubation depth (cm)	
Number of intubation attempts		
Intubation time (min)*		
Whether to re-intubation		
Heart rate and blood pressure immediately after intubation (indicating time)		
Heart rate and blood pressure immediately after lateral positioning (indicating time)		
FOB usage	Total	
	1st use time and reason	
	2nd use time and reason	
	3rd use time and reason	
DLT malposition (definition is that the DLT is moved to correct its position by more than 1.0 cm)	Total	
	1st malpostion time, distance and processing	

	2nd malpostion time, distance and processing	
	3rd malpostion time, distance and processing	
Changes in heart rate and blood pressure and treatment measures		
Occurrence frequency and minimum value of hypoxemia		
Whether to change the surgical method (including lymph node dissection and scope) and the final method		
Whether to place closed drainage		
Postoperative analgesia mode, drug type and dose		

CRF: case record form; min: minute; ml: milliliter; DLT: double-lumen tube; cm: centimeter; *Defined as the time from the use of the video-laryngoscope to confirm the correct position of the DLT by using the FOB; FOB: fiberoptic bronchoscopy; 1st: first; 2nd: second; 3rd: third. If the number of FOB uses or DLT malposition is greater than 3, then another intraoperative CRF table is taken.

Postoperative CRF Name:______ Inpatient number:_____

Extubation time		
Whether pharyngeal discomfort and degree after extubation		
PACU retention time (min)		
PACU score		
QoR-15 score		
Complications of the upper arm of lateral compression		
Postoperative antibiotic use		
Other complications	Sore throat and hoarseness	
	lung infection	
	Incision infection	
	Secondary operation	
	Pulmonary embolism	
	Acute respiratory failure	
	Others	
Satisfaction survey (1-10 points)	Surgeon	
	Nurse	
	Patient	
Hospitalization days and expenses		

CRF: case record form; min: minute; PACU: postanesthesia care unit; QoR-15: Quality of Recovery-15.
