# Supplementary material for: Effect of intubation in the lateral position under general anesthesia induction on the position of double-lumen tube placement in patients undergoing unilateral video-assisted thoracic surgery: study protocol for a prospective, single-center, parallel group, randomized, controlled trial
Source: Trials. 2023 Jan 29;24:67. doi: 10.1186/s13063-023-07075-9 (PMC9884328; doi:10.1186/s13063-023-07075-9)
Supplement: Supplementary file 2 — Additional file 2. QoR-15 score. [file 13063_2023_7075_MOESM2_ESM.pdf]

QoR-15 Patient Survey

Date: _ _ / _ _ / _ _ Name: __________ Study #: ____________

Preoperative ☐ Postoperative ☐

PART A

How have you been feeling in the last 24 hours?

(0 to 10, where: 0 = none of the time [poor] and 10 = all of the time [excellent])

1. Able to breathe easily	None of the time	0	1	2	3	4	5	6	7	8	9	10	All of the time
2. Been able to enjoy food	None of the time	0	1	2	3	4	5	6	7	8	9	10	All of the time
3. Feeling rested	None of the time	0	1	2	3	4	5	6	7	8	9	10	All of the time
4. Have had a good sleep	None of the time	0	1	2	3	4	5	6	7	8	9	10	All of the time
5. Able to look after personal toilet and hygiene unaided	None of the time	0	1	2	3	4	5	6	7	8	9	10	All of the time
6. Able to communicate with family or friends	None of the time	0	1	2	3	4	5	6	7	8	9	10	All of the time
7. Getting support from hospital doctors and nurses	None of the time	0	1	2	3	4	5	6	7	8	9	10	All of the time
8. Able to return to work or usual home activities	None of the time	0	1	2	3	4	5	6	7	8	9	10	All of the time
9. Feeling comfortable and in control	None of the time	0	1	2	3	4	5	6	7	8	9	10	All of the time
10. Having a feeling of general well-being	None of the time	0	1	2	3	4	5	6	7	8	9	10	All of the time

PART B

Have you had any of the following in the last 24 hours?

(10 to 0, where: 10 = none of the time [excellent] and 0 = all of the time [poor])

11. Moderate pain	None of the time	10	9	8	7	6	5	4	3	2	1	0	All of the time
12. Severe pain	None of the time	10	9	8	7	6	5	4	3	2	1	0	All of the time
13. Nausea or vomiting	None of the time	10	9	8	7	6	5	4	3	2	1	0	All of the time
14. Feeling worried or anxious	None of the time	10	9	8	7	6	5	4	3	2	1	0	All of the time
15. Feeling sad or depressed	None of the time	10	9	8	7	6	5	4	3	2	1	0	All of the time

Thank you for your assistance.

Please check that all questions have been answered.

If you have any questions, please contact: Siping Hu 137-3820-6677.
