# Supplementary material for: Effect of intubation in the lateral position under general anesthesia induction on the position of double-lumen tube placement in patients undergoing unilateral video-assisted thoracic surgery: study protocol for a prospective, single-center, parallel group, randomized, controlled trial
Source: Trials. 2023 Jan 29;24:67. doi: 10.1186/s13063-023-07075-9 (PMC9884328; doi:10.1186/s13063-023-07075-9)
Supplement: Supplementary file 3 — Additional file 3. Informed consent form. [file 13063_2023_7075_MOESM3_ESM.pdf]

Informed consent

Version No: 2.0 Version Date: May 5, 2022

Dear patient:

We invite you to take part in a clinical study entitled "Effect of intubation in the lateral position under general anesthesia induction on the position of double-lumen tube placement in patients undergoing unilateral video-assisted thoracic surgery: a prospective randomized controlled study". Before you decide whether to participate in this study, please read the following carefully. It can help you understand the purpose, the procedure and duration of the study, and the possible benefits, risks and inconveniences after participating in the study. If you have any questions or don't understand, please consult the researcher (doctor) until you fully understand. You can also discuss with your family, relatives, friends, etc. to help you make a decision that is in your interest.

The following is an introduction to this study:

1. Study background and purpose

At present, the number of thoracic surgery is still increasing, especially under the influence of the severe COVID-19 epidemic. Video-assisted thoracic surgery (VATS) has many advantages, such as less injury, wider surgical field and faster postoperative recovery. It has been widely used in clinic, and its key is lung isolation and one-lung ventilation technology. Therefore, double-lumen tube (DLT) is routinely used in endoscopic surgery to meet the needs of one-lung ventilation. However, in clinical practice, it is found that DLT often shifts after intubation. Related literature reports that the incidence of catheter shift is as high as 39.5%, which will lead to inaccurate location of DLT, failure of lung isolation and inability of one-lung ventilation. In this study, the patients undergoing elective thoracoscopic surgery for DLT intubation under general anesthesia in our hospital were taken as the study object, and the influence of intubation on DLT malposition rate after induction of general anesthesia in lateral position was evaluated, and clinical evidence was provided for the feasibility of this intervention measure. The study results provided a new method for DLT intubation under general anesthesia, thus reducing the related risks caused by DLT malposition, posture change after anesthesia and repeated use of fiberoptic bronchoscope (FOB). We also explored the vital signs and the incidence of hypoxemia during the operation after

intubation in lateral position, so as to ensure the safety and stability of patients during the operation. At the same time, we pay attention to the complications of contralateral upper arm and Quality of Recovery-15 (QoR-15) score, which is more in line with the concept of accelerated rehabilitation surgery (ERAS).

Principal in charge:

Hu Si-Ping, Master of Anesthesiology of Zhejiang University, Chief Physician of Anesthesiology Department of Huzhou Central Hospital, Master Supervisor of Medical College of Zhejiang University and Huzhou University, National Member of Brain and Vascular Branch of China Cardiovascular Anesthesiology Society, and Member of Cardiothoracic Anesthesiology Group of Zhejiang Province, specializes in intraoperative management and postoperative analgesia of cardiothoracic surgery.

Project team members:

Liu He: Deputy Chief Physician, PhD; Shen Qi-Bin: Chief Physician of Cardiothoracic Surgery; Chen Juan-Li: Chief Nurse; Tong Fei: Attending Physician; Hu Yong-He: Resident; Zhang Zhen-Duo: Resident; Liu Fei-Fan: Resident; Tang Ya-Wen: Resident; Zhang Qin: Resident; Zhang Xi and Wang Dong-Xu: graduate student in Anesthesiology;

2. Specific procedures and processes

Methods General intravenous anesthesia is used for anesthesia. Five-lead electrocardiogram (ECG), noninvasive arterial blood pressure, blood oxygen saturation will be monitored after entering the room. Routine supine intubation group: before anesthesia induction, the patients take the routine supine position, and after anesthesia induction, the left-sided DLT will be intubated under laryngoscope, and FOB was will be used to observe and adjust the DLT position to complete the DLT positioning; The medical staff adjust the patient's position to the lateral position meeting the operation requirements, and confirm the position with FOB again. Lateral intubation group: before anesthesia induction, patients will be assisted to a comfortable lateral position that meets the requirements of surgery. After anesthesia induction, the left-sided DLT will be intubated under laryngoscope, and FOB will be used to confirm and adjust the position of DLT, so as to complete the positioning of DLT. The preoperative general information, airway evaluation, DLT type, intubation depth, intubation time, whether to re-intubation, intubation times, intraoperative vital signs, DLT malposition, the frequency and duration of re-adjustments under FOB,

hypoxemia frequency, operation duration, operation type, extubation time, the degree of pharyngeal discomfort, etc. of the two groups will be recorded. The complications and the QoR-15 will be followed up 24 hours after the operation. The number of follow-up is one, which took about 5 minutes. It will be mainly used to fill out the QoR-15 and the complications.

3. What do you need to do if you take part in the study

If you agree to participate in our study, please sign the informed consent form one day before operation. We will routinely monitor your vital signs and give you mask oxygen before anesthesia. Before induction, open the sealed envelope with the random number table, and randomly assign you to the routine intubation group or the lateral intubation group according to the digital information. Please cooperate with relevant operations. If in doubt, we will give you a detailed explanation.

4. The possible benefits of participating in this study

The medical benefits that this study may bring to you: this study will probably reduce the related risks caused by DLT malposition, posture change after anesthesia, and repeated use of FOB. Moreover, the intraoperative hemodynamics may be more stable and the incidence of hypoxemia will be reduced after intubation in lateral position, thus ensuring a safer and more stable operation period. At the same time, this study will probably reduce the postoperative complications, the hospitalization days and medical expenses.

5. Possible adverse reactions, risks, prevention and treatment measures of participating in this study

If lateral intubation fails with try lateral intubation ≥3 times or intubation time ≥3 minutes, after ensuring the safety of patients, then change to conventional supine intubation. Relevant literature has shown that lateral intubation is a successful and safe intubation method, so the possibility of lateral intubation failure leading to serious consequences is relatively small. The possible adverse reactions of lateral DLT intubation are the same as those of conventional intubation, such as tooth loss, oral mucosa injury, airway injury, pharyngeal discomfort, etc. Therefore, during the implementation of the study, the operators are specially trained and skilled in intubation skills. Before intubation, they will fully evaluate the patient's airway condition, predict the difficulty of intubation, and formulate corresponding plans to prevent it. In case of emergency, contact Hu Si-Ping, the project leader, at 13739206677.

6. Description of expenses

This study does not need additional procedures, and will not increase the additional expenses of the subjects. It is expected to reduce the total medical expenses of patients during hospitalization.

7. Alternative scheme

This study is an intervention study, which will not affect the normal diagnosis and treatment activities of the subjects. If you don't take part in this study, the alternative plan is: conventional DLT intubation after anesthesia induction.

8. Your rights

Whether to participate in this study depends entirely on your willingness. You can refuse to take part in this study, or withdraw from it at any time during the study without any reason, which will not affect your relationship with the doctor, and will not affect the loss of your medical treatment or other interests. If you have any questions about this study or have any problems in the study process, please contact Hu Si-Ping, the researcher of this project, at 13739206677.

9. Confidentiality of your personal information

Your medical records (including study medical records and, physical and chemical examination reports, etc.) will be kept in the hospital as required. Except researchers, Ethics Committee, supervision, inspection, pharmaceutical administration department and other related personnel, other personnel unrelated to the study have no right to consult your medical records without permission. The public report of this study result will not disclose your personal information. We will make every effort to protect the privacy of your personal medical data to the extent permitted.

10. Termination of participation in the study

Your participation in this study may be terminated due to the following reasons:

- You did not follow the doctor's advice of the study doctor.
- You have a serious situation that may require treatment.
- The study doctor believes that it is best for your health to terminate the study.

11. Ethics Committee

This study has been reported to the Medical Ethics Committee of Huzhou Central Hospital and approved by the Committee. During the study process, you can contact the Medical Ethics

Committee of Huzhou Central Hospital for matters related to ethics and rights.

Tel: 0572-2709719; Email address:hzszxyyll@163.com

Researcher statement:

I confirmed that I had explained the details of this study to the patient, including his rights, possible benefits and risks, and answered his (and/or guardian/witness) questions. He (and/or guardian/witness) said that he understood my instructions and explanations. I have given him (and/or guardian/witness) a copy of the signed informed consent form.

Signature of researcher: _ _ _ _ _ _ _ _ _ _ _ _ _ _ _ _

Contact number of researcher: _ _ _ _ _ _ _ _ _ _ _ _ _ _ _ _

Date of conversation: _ _ _ _ _ _ _ _ _ _ _ _ _ _ _ _

The subject (or guardian/witness) declares that:

I confirm that I have read the informed consent form of this study. The researcher explained the relevant contents to me in detail, answered my related questions, and made me understand the terms and conditions. I also confirmed that if I did not participate in this study or quit at any time in the middle of the study, it would not affect my diagnosis and treatment activities and doctor-patient relationship, or cause other interests damage. My personal information will not be disclosed in the public reports that I know the results of this study. I have plenty of time to think about it. After careful consideration, I decided to accept the treatment (study) methods in this study, and agreed to use my relevant study data and information for the public reports related to the results of this study.

Subject's signature: _ _ _ _ _ _ _ _ _ _ _ _ _ _ _ _ Signature date: _ _ _ _ _ _ _ _ _ _ _ _ _ _ _ _

Contact number of the subject: _ _ _ _ _ _ _ _ _ _ _ _ _ _ _ _

If the subject has insufficient self-knowledge or incapability, his guardian should sign it.

Guardian's signature: _ _ _ _ _ _ _ _ _ _ _ _ _ _ _ _ _ Signature date: _ _ _ _ _ _ _ _ _ _ _ _ _ _ _ _ _

Contact number of guardian: _ _ _ _ _ _ _ _ _ _ _ _ _ _ _ _ _

If the subject or his guardian can't read or write, the witness's signature is required.

Signature of witness: _ _ _ _ _ _ _ _ _ _ _ _ _ _ _ _ _ Signature date: _ _ _ _ _ _ _ _ _ _ _ _ _ _ _ _

Witness contact number: _ _ _ _ _ _ _ _ _ _ _ _ _ _ _ _ _
